# AP2/ERF Family Transcription Factors ORA59 and RAP2.3 Interact in the Nucleus and Function Together in Ethylene Responses

**DOI:** 10.3389/fpls.2018.01675

**Published:** 2018-11-19

**Authors:** Na Young Kim, Young Jin Jang, Ohkmae K. Park

**Affiliations:** Department of Life Sciences, Korea University, Seoul, South Korea

**Keywords:** *Arabidopsis thaliana*, ORA59, RAP2.3, ethylene response factor, ethylene, *Pectobacterium carotovorum*, disease resistance, plant immunity

## Abstract

The gaseous plant hormone ethylene is a key signaling molecule regulating plant growth, development, and defense against pathogens. Octadecanoid-responsive arabidopsis 59 (ORA59) is an ethylene response factor (ERF) transcription factor and has been suggested to integrate ethylene and jasmonic acid signaling and regulate resistance to necrotrophic pathogens. Here we screened for ORA59 interactors using the yeast two-hybrid system to elucidate the molecular function of ORA59. This led to the identification of RELATED TO AP2.3 (RAP2.3), another ERF transcription factor belonging to the group VII ERF family. In binding assays, ORA59 and RAP2.3 interacted in the nucleus and showed ethylene-dependent nuclear localization. ORA59 played a positive role in ethylene-regulated responses, including the triple response, featured by short, thick hypocotyl and root, and exaggerated apical hook in dark-grown seedlings, and resistance to the necrotrophic pathogen *Pectobacterium carotovorum*, as shown by the increased and decreased ethylene sensitivity and disease resistance in ORA59-overexpressing (*ORA59OE*) and null mutant (*ora59*) plants, respectively. In genetic crosses, *ORA59OE rap2.3* crossed lines lost ORA59-mediated positive effects and behaved like *rap2.3* mutant. These results suggest that ORA59 physically interacts with RAP2.3 and that this interaction is important for the regulatory roles of ORA59 in ethylene responses.

## Introduction

During evolution, plants have become equipped with defense mechanisms that enable them to survive against environmental stresses. The survival strategies of plants include the timely production of phytohormones that play key roles in regulating plant defense (Gupta et al., 2017). Salicylic acid (SA), jasmonic acid (JA), and ethylene are major hormones regulating the defense against pathogens (Glazebrook, 2005; Kwon et al., 2009; Pieterse et al., 2012). Other hormones such as abscisic acid (ABA), auxin, gibberellin (GA), cytokinin, and brassinosteroid have also been implicated in defense responses (Siemens et al., 2006; Wang et al., 2007; Asselbergh et al., 2008; Navarro et al., 2008; Shan et al., 2008). SA is generally associated with resistance to biotrophic pathogens such as *Pseudomonas syringae* and *Hyaloperonospora arabidopsidis*, whereas JA and ethylene trigger resistance to necrotrophic pathogens such as *Alternaria brassicicola* and *Botrytis cinerea* (Pieterse et al., 1996; Penninckx et al., 1998; Glazebrook, 2005). Hormones or signaling molecules function through signaling pathways that are interconnected to form a complex network. Numerous reports have described both antagonistic and synergistic interactions between SA and JA/ethylene pathways (Kunkel and Brooks, 2002; Spoel and Dong, 2008). Whereas ethylene and JA signaling often interact synergistically, there is antagonism between SA and JA/ethylene (Penninckx et al., 1998; Thomma et al., 1998; Koornneef et al., 2008; Kim et al., 2013). This may be the outcome of evolution towards reducing the fitness cost, enabling plants to prioritize either the SA or JA/ethylene pathway depending on the lifestyle of invading pathogens.

The gaseous hormone ethylene plays pivotal roles in plant growth, development, and stress responses (Wang et al., 2002; van Loon et al., 2006; Cho and Yoo, 2009). Ethylene initiates a signaling cascade when bound to ethylene receptor family members in the endoplasmic reticulum (ER): ethylene resistance 1 (ETR1), ETR2, ethylene insensitive 4 (EIN4), ethylene response sensor 1 (ERS1), and ERS2 in Arabidopsis (Chang et al., 1993; Hua and Meyerowitz, 1998; Hua et al., 1998; Sakai et al., 1998). In the absence of ethylene, ethylene receptors act as negative regulators and activate constitutive triple response 1 (CTR1), a Raf-like serine/threonine kinase that phosphorylates an ER-located membrane protein EIN2 to repress ethylene signaling (Kieber et al., 1993). The presence of ethylene switches off CTR1, leading to activation of positive regulators EIN2 and EIN3 (Chao et al., 1997). EIN2 is cleaved, releasing the C-terminal fragment, which moves to the nucleus and stabilizes EIN3 and EIN3-like 1 (EIL1) by downregulating EIN3-binding F-box protein 1 (EBF1) and EBF2 required for EIN3/EIL1 degradation (Guo and Ecker, 2003; Potuschak et al., 2003; Gagne et al., 2004; Ju et al., 2012; Qiao et al., 2012; Wen et al., 2012). EIN3 and EIL1 further regulate the expression of the ethylene response factor (ERF) family transcription factors, belonging to the APETALA2 (AP2)/ERF superfamily (Solano et al., 1998).

Many reports have demonstrated the importance of ERFs in crosstalk among ethylene, JA, and SA for regulating disease resistance (Caarls et al., 2017). ERF1 and octadecanoid-responsive arabidopsis 59 (ORA59) belong to the group IX ERF family and have been suggested to act as integrators of ethylene and JA pathways (Lorenzo et al., 2003; Pré et al., 2008). The expression of JA/ethylene-responsive pathogenesis-related genes plant defensin 1.2 (PDF1.2) and basic chitinase (b-CHI) was induced synergistically by JA and ethylene, depending on ERF1 and ORA59 (Pré et al., 2008). In binding analyses, ERF1 and ORA59 directly bound to GCC boxes in the PDF1.2 promoter, resulting in a synergistic response to JA and ethylene (Zarei et al., 2011). *ERF1* and *ORA59* themselves were also activated by JA and ethylene in a synergistic manner. This JA/ethylene-dependent expression was abolished in JA-insensitive *coronatine insensitive 1* (*coi1*) or ethylene-insensitive *ein2* mutants (Penninckx et al., 1998; Lorenzo et al., 2003; Pré et al., 2008). Overexpression of *ERF1* and *ORA59* enhanced resistance to necrotrophic pathogens such as *Botrytis cinerea*, indicating that ERF1 and ORA59 are major components in JA/ethylene-regulated defense responses (Berrocal-Lobo et al., 2002; Pré et al., 2008). Additionally, ORA59 has been evaluated as a target of SA-mediated antagonism of JA signaling (Van der Does et al., 2013). SA suppressed JA-responsive gene expression by downregulating ORA59 accumulation. In agreement with this, *ORA59* overexpression in plants counteracted the SA-mediated antagonistic effect on JA-mediated *PDF1.2* expression (Leon-Reyes et al., 2010; Van der Does et al., 2013; He et al., 2017).

Here an ERF family transcription factor, related to AP2.3 (RAP2.3), was identified as an ORA59-interacting protein by yeast two-hybrid (Y2H) screening. We examined the interactions and functions of ORA59 and RAP2.3 in response to ethylene. ORA59 positively regulated the ethylene-triggered triple response and resistance to the necrotrophic pathogen *Pectobacterium carotovorum* (also called *Erwinia carotovora*), which depended on RAP2.3. These results suggest that ORA59 interacts with RAP2.3 to regulate ethylene-dependent responses.

## Materials and Methods

### Plant Materials and Growth Conditions

*Arabidopsis thaliana* (ecotype Columbia, Col-0) plants were grown at 23°C under long-day conditions (16-h light/8-h dark cycle) for general growth and under short-day conditions (8-h light/16-h dark cycle) for pathogen infection and hormone treatments. The following plant lines were used in this study: *ein2-1* (Yoo et al., 2008), *ein3-1 eil1-1* (Alonso et al., 2003), *EIN3OE* (Chao et al., 1997), *rap2.3-2* (Marín-de la Rosa et al., 2014), and *erfVII* (Marín-de la Rosa et al., 2014). *ora59-1* (GABI_CS405772) and *TPT_RAP2.3* (GK-210F05.02) lines were obtained from the Arabidopsis Biological Resource Center (Columbus, OH, United States) and Nottingham Arabidopsis Stock Centre (Nottingham, United Kingdom), respectively. To generate *ORA59OE* plants, the full-length cDNA of *ORA59* was amplified and cloned into the binary vector pCAMBIA1300 under the control of the cauliflower mosaic virus (CaMV) 35S promoter. The construct was transformed into Arabidopsis plants using the floral dip method (Clough and Bent, 1998). Transformants were selected on 1/2 MS media containing 10 μg/mL hygromycin and homozygous T3 seeds were used for experiments. *ORA59OE* and *ora59-1* lines were crossed with *rap2.3-2* and *erfVII* mutants, and homozygous lines were confirmed by PCR and sequence analysis using gene-specific primers (Supplementary Table S1).

### Plant Treatments

For pathogen infection, 4-week-old plants grown under short-day conditions were used (Lee et al., 2017). *P. carotovorum* was cultured in L-medium containing 100 μg/mL ampicillin at 28°C for 1 day. Leaves were infiltrated with 10 μL of NaCl (0.9%) or a bacterial suspension (10^4^–10^5^ cfu/mL) and incubated for 1 day before analysis of disease development and gene expression. For hormone treatments, 4-week-old plants grown under short-day conditions were sprayed with ethephon (ET; 1.5 mM), salicylic acid (SA; 1 mM), methyl jasmonate (MeJA; 50 μM), abscisic acid (ABA; 10 μM), and gibberellin (GA; 10 μM) dissolved in 0.1% (v/v) dimethyl sulfoxide (DMSO). The treated plants were maintained at 100% humidity for the indicated times.

### Phenotypic Analysis of Etiolated Seedlings

Phenotypes of 1-aminocyclopropane-carboxylic acid (ACC)-treated etiolated seedlings, the so-called triple response, were analyzed as previously described (Yoo et al., 2008). Sterilized seeds were plated on 1/2 MS media containing 1% sucrose alone or supplemented with 10 μM ACC. For *pER8* and *TPT_RAP2.3* lines, media was additionally supplemented with 5 μM β-estradiol. Plates were wrapped in foil to maintain the dark conditions and kept for 4 days at 23°C.

### Yeast Two-Hybrid Screening

Total RNAs were isolated from 6-week-old Col-0 plants treated with 1.5 mM ethephon for 24 h and used to construct a Mate & Plate^TM^ library in accordance with the protocol of the Matchmaker^TM^ Gold Yeast Two-Hybrid System (CLONTECH, Mountain View, CA, United States). Y2H screening was performed according to the manufacturer’s instructions. To test for the interaction of ORA59 with RAP2.3, *ORA59* and *RAP2.3* were cloned into the pGADT7 and pGBKT7 vectors (CLONTECH). The yeast strain AH109 was co-transformed with the constructs, grown on synthetic dextrose (SD)/-Leu-Trp media, and then transferred to SD/-Ade-His-Leu-Trp media. Transactivation activity was further evaluated based on the activity of α-galactosidase on SD/-Ade-His-Leu-Trp media containing 40 mg/L of X-α-Gal.

### Gene Expression Analysis

Total RNAs were extracted from 4-week-old plants grown under short-day conditions using TRIsure reagent (BIOLINE, London, United Kingdom) and used to synthesize first-strand cDNAs with a PrimeScript^TM^ RT reagent Kit (TAKARA, Shiga, Japan). Quantitative real-time PCR was performed using KAPA SYBR FAST qPCR master mix (KAPA Biosystems, Wilmington, MA, United States) with gene-specific primers (Supplementary Table S1) on a LightCycler 480 system (ROCHE, Basel, Switzerland) according to the manufacturer’s protocol. The expression of tested genes was normalized to the constitutive expression level of *ACTIN1* and calculated using the LinRegPCR software (Heart Failure Research Center). Experiments were repeated at least three times with biologically independent samples.

### GST Pull-Down Assay

ORA59 and RAP2.3 were tagged with glutathione *S*-transferase (GST) or His at the C-terminus and recombinant proteins were affinity-purified with glutathione sepharose 4B beads (GE Healthcare, Little Chalfont, United Kingdom) or Ni-NTA chelating agarose CL-6B (PEPTRON, Daejeon, Korea), according to the manufacturer’s instructions. A pull-down assay was performed as previously described (Lee et al., 2017), with some modifications. GST- or ORA59-GST (10 μg) was incubated with glutathione sepharose 4B beads for 2 h in binding buffer (25 mM Tris-HCl, pH 8.0, 100 mM NaCl, 1% Triton X-100, and 1 mM DTT) at 4°C. RAP2.3-His (10 μg) was added and reacted for 2 h at 4°C. Bound proteins were eluted by boiling in 6× SDS loading buffer (350 mM Tris-HCl, pH 6.8, 10% SDS, 30% glycerol, 5% β-mercaptoethanol, and 0.02% bromophenol blue), separated by SDS-PAGE, and visualized by immunoblotting using anti-GST and anti-His antibodies (SANTA CRUZ, Dallas, TX, United States).

### BiFC and Subcellular Localization

For BiFC, *bZIP63*, *ORA59*, and *RAP2.3* were cloned into the pUC-SPYNE and pUC-SPYCE vectors for the expression of fusion proteins bZIP63-SPYNE, bZIP63-SPYCE, ORA59-SPYNE, and RAP2.3-SPYCE. For subcellular localization, *ORA59* and *RAP2.3* were cloned into the pUC-GFP and pUC-RFP vectors for the expression of fusion proteins ORA59-GFP, RAP2.3-GFP, and RAP2.3-RFP. GFP, RFP, and split YFP (SPYNE and SPYCE) were fused to the C-terminus of ORA59 and RAP2.3. The pUC-NLS-RFP vector was used for a nuclear marker. Arabidopsis protoplasts were transfected with these constructs and incubated for 20–24 h for protein expression and then treated with 0.1% DMSO (mock) or 1.5 mM ET for 4 h. Fluorescence signals were visualized using a confocal microscope (LSM 700; ZEISS, Oberkochen, Germany).

### Trypan Blue Staining

Trypan blue staining was performed as previously described (Bowling et al., 1997). Leaves were immersed in lactophenol blue solution (Sigma-Aldrich, St. Louis, United States) and reacted for overnight. Stained leaves were placed in a 60% chloral hydrate solution and finally equilibrated with 50% glycerol.

## Results

### ORA59 Physically Interacts With RAP2.3

To identify proteins that interact with ORA59, we performed Y2H screening using a cDNA library prepared from ethephon-treated Arabidopsis plants. Full-length ORA59 was fused to the GAL4 activation domain (AD) and tested for autoactivation before screening. Yeast cells transformed with the AD-ORA59 construct alone grew on selective media lacking adenine, histidine, leucine, and tryptophan (SD/-AHLT), indicating that ORA59 can autoactivate the reporter gene (Supplementary Figure S1). To prevent autoactivation, we made truncations at the N- or C-terminal regions of ORA59. The truncated form of ORA59 with N-terminal 60 amino acids deleted (ORA59Δ1-60) showed no autoactivation, and therefore, was used as a bait construct. Y2H screening led to the isolation of 12 proteins as potential ORA59 interactors (Table 1); among these, another ERF member RAP2.3 was chosen for further analysis.

**Table 1:** Potential ORA59 interactors identified in the Y2H screen.

AGI code	Gene name	Description
AT5G42100	BG_PPAP	β-1,3-glucanase, plasmodesmal (Pd)-associated membrane protein involved in plasmodesmal callose degradation
AT5G51880		2-oxoglutarate (2OG) and Fe (II)-dependent oxygenase superfamily protein
AT1G08520	ALB1	CHLD subunit of the Mg-chelatase enzyme involved in chlorophyll biosynthesis
AT1G68010	HPR	Hydroxypyruvate reductase
AT3G12780	PGK1	Phosphoglycerate kinase 1
AT3G16770	RAP2.3	Member of the ethylene response factor (ERF) subfamily B-2 of the plant specific ERF/AP2 transcription factor family
AT5G58070	TIL	Temperature-induced lipocalin TIL 1
AT4G05320	UBQ10	Polyubiquitin 10, the highly conserved 76-amino acid protein ubiquitin that is covalently attached to substrate proteins targeting most for degradation
AT3G26520	TIP2	Gamma tonoplast intrinsic protein 2
AT5G16050	GRF5	General regulatory factor 5, GF14 upsilon chain, a 14-3-3 gene family member
AT2G41100	TCH3	Calmodulin-like protein, with six potential calcium binding domains
AT2G30950	VAR2	FtsH extracellular protease family, metalloprotease that functions in thylakoid membrane biogenesis

**FIGURE 1:**
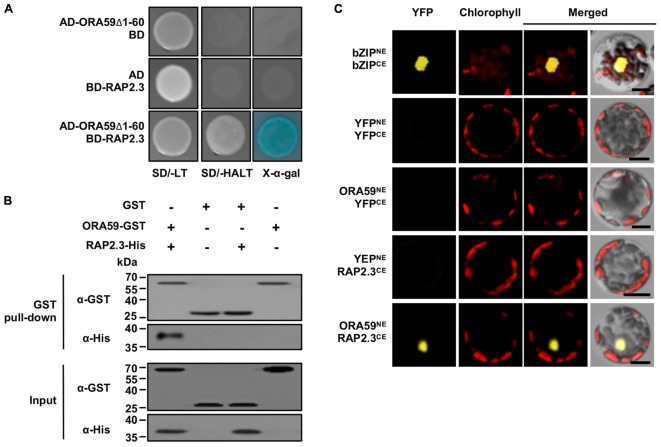
Physical interaction of ORA59 with RAP2.3. **(A)** Yeast two-hybrid assay. ORA59 with N-terminal 60 amino acids deleted (ORA59Δ1-60) and full-length RAP2.3 were fused with GAL4 AD and BD, respectively. Their interactions were tested on selective media SD/-AHLT and in the presence of X-α-Gal. **(B)** *In vitro* GST pull-down assay. GST or ORA59-GST was incubated with RAP2.3-His and precipitated with glutathione sepharose 4B beads. Proteins were detected by immunoblotting with anti-GST and anti-His antibodies. Input shows 1% of the amount used in binding reactions. WB, western blotting. **(C)** BiFC assay. YFP^NE^, YFP^CE^, and their fusion proteins bZIP63^NE^, bZIP63^CE^, ORA59^NE^, and RAP2.3^CE^ were expressed in Arabidopsis protoplasts as indicated. YFP fluorescence signals were visualized under a confocal microscope. Bars, 10 μm. Experiments were repeated three times with similar results.

Because the clones selected from Y2H screening had partial sequences, the entire RAP2.3 cDNA was cloned and re-tested for the interaction with ORA59 in the Y2H system (Figure 1A). Co-expression of ORA59Δ1-60 fused to the GAL4 AD and RAP2.3 to the GAL4 DNA-binding domain (BD) enabled yeast cells to grow on SD/-AHLT media and turn blue in the presence of X-α-Gal, indicating a direct interaction of ORA59 and RAP2.3 in yeast. We performed additional binding analyses. The *in vitro* interaction was verified in a GST pull-down assay using C-terminal GST-tagged ORA59 (ORA59-GST) and His-tagged RAP2.3 (RAP2.3-His) recombinant proteins (Figure 1B). The *in vivo* interaction was assessed by bimolecular fluorescence complementation (BiFC) assays. ORA59 fused with the N-terminal part of YFP (ORA59^*NE*^) and RAP2.3 fused with the C-terminal part of YFP (RAP2.3^*CE*^) were transiently expressed in Arabidopsis protoplasts. Their co-expression resulted in nuclear fluorescence signals (Figure 1C), suggesting that ORA59 interacts with RAP2.3 in the nucleus of plant cells.

### Expression Analysis of *ORA59* and *RAP2.3*

**FIGURE 2:**
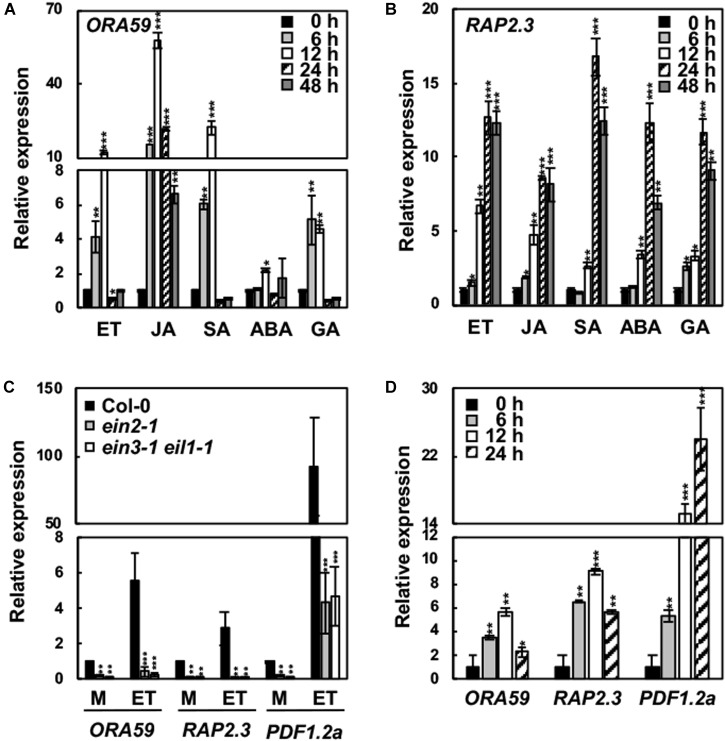
Hormone responses of *ORA59* and *RAP2.3* expression. **(A,B)** Expression of *ORA59* **(A)** and *RAP2.3* **(B)** in response to hormone treatments. **(C)** Expression of *ORA59* and *RAP2.3* in *ein2* and *ein3 eil1* backgrounds. **(D)** Expression of *ORA59* and *RAP2.3* in response to *P. carotovorum* inoculation. Four-week-old plants were treated with ET (1.5 mM), MeJA (50 μM), SA (1 mM), ABA (10 μM), or GA (10 μM) for the indicated times in **(A,B)**, with 0.1% DMSO (mock) or ET (1.5 mM) for 6 h in **(C)**, and with *P. carotovorum* (10^4^ cfu/mL) for the indicated times in **(D)**. The values represent means ± SD from three independent experiments. Asterisks indicate significant differences from 0 h treatment **(A,B,D)** and Col-0 **(C)** (*t-*test; ^∗∗^*P* < 0.01; ^∗∗∗^*P* < 0.001).

We examined the responses of *ORA59* and *RAP2.3* expression to hormones. For this, Col-0 plants were treated with SA, MeJA, ET, ABA, and GA. Consistent with the previous report (Pré et al., 2008), *ORA59* expression was induced by MeJA, ET, and SA, as well as by GA to a slightly lower level (Figures 2A,B). However, all these hormones increased the expression of *RAP2.3*. Basal and ET-induced expression of *ORA59* and *RAP2.3* was largely decreased in *ein2* and *ein3 eil1* mutant plants (Figure 2C). These results indicate that *ORA59* and *RAP2.3* expression is regulated by ethylene signaling components such as EIN2 and EIN3/EIL1. We further analyzed *ORA59* and *RAP2.3* expression in response to pathogen infection. When inoculated with the necrotrophic bacterial pathogen *P. carotovorum*, Col-0 plants strongly expressed both *ORA59* and *RAP2.3* (Figure 2D), suggesting that ORA59 and RAP2.3 may function together in immune responses.

### Nuclear Localization of ORA59 and RAP2.3 Is Promoted by ET Treatment

**FIGURE 3:**
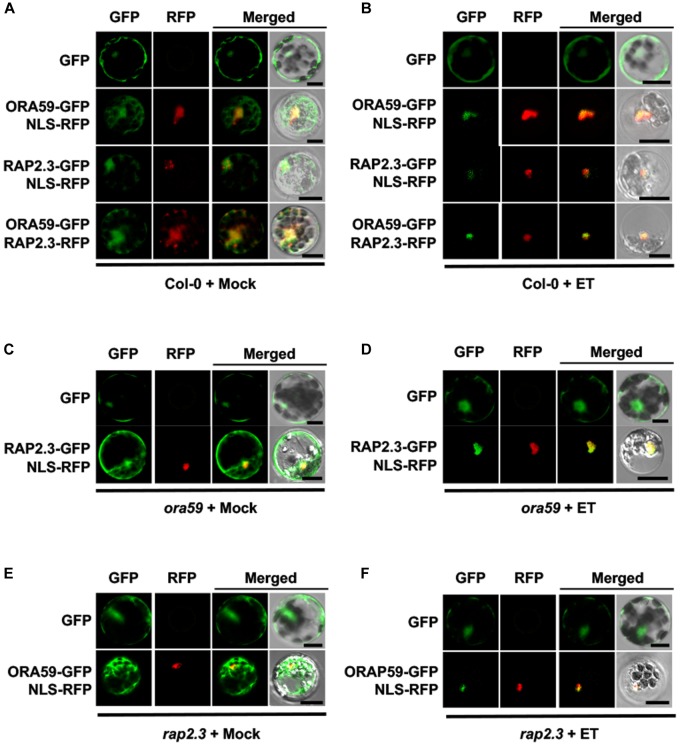
ET-dependent nuclear co-localization of ORA59 and RAP2.3. **(A,B)** Subcellular localization of ORA59 and RAP2.3 in Col-0 protoplasts treated with 0.1% DMSO (mock) **(A)** or 1.5 mM ET **(B)** for 4 h. **(C,D)** Subcellular localization of ORA59 in *rap2.3* protoplasts treated with 0.1% DMSO (mock) **(C)** or 1.5 mM ET **(D)** for 4 h. **(E,F)** Subcellular localization of RAP2.3 in *ora59* protoplasts treated with 0.1% DMSO (mock) **(E)** or 1.5 mM ET **(F)** for 4 h. ORA59-GFP, RAP2.3-GFP, RAP2.3-RFP, and NLS-RFP were expressed in Arabidopsis protoplasts as indicated. Fluorescence signals were visualized under a confocal microscope. Bars, 10 μm. Experiments were repeated three times with similar results.

Subcellular localization of ORA59 and RAP2.3 was determined with or without ET treatment. Arabidopsis Col-0 protoplasts were transfected with GFP- or red fluorescent protein (RFP)-fused ORA59 and RAP2.3 constructs. ORA59 and RAP2.3 fluorescence signals were both detected in the cytosol and nucleus, showing substantial co-localization in these compartments (Figure 3A). After ET treatment, ORA59 and RAP2.3 proteins were mostly localized to the nucleus (Figure 3B). This suggests that the transcriptional activity of ORA59 and RAP2.3 is regulated through nuclear targeting, which is regulated by ethylene signaling. However, ET-dependent nuclear localization of ORA59 and RAP2.3 was not affected in *rap2.3* and *ora59* mutant backgrounds, respectively (Figures 3C–F), indicating that the interaction of ORA59 and RAP2.3 is not required for nuclear translocation.

### ORA59 Positively Affects the Triple Response in an RAP2.3-Dependent Manner

For functional analysis, mutant and transgenic Arabidopsis lines for *ORA59* and *RAP2.3* were prepared. We obtained *ora59-1* mutant (Supplementary Figures S2A–C) and *rap2.3-2* mutant (Marín-de la Rosa et al., 2014; Supplementary Figure S3A), and generated transgenic plants overexpressing *ORA59* under the control of the CaMV 35S promoter (*ORA59OE*) (Supplementary Figure S2C). A transgenic transplanta (TPT) line conditionally overexpressing *RAP2.3* under the control of the β-estradiol-inducible promoter (*TPT_RAP2.3*) was obtained (Coego et al., 2014) and tested together with a vector control line (*pER8*) (Supplementary Figure S3A). RAP2.3 is a member of the group VII ERF family, and genetic redundancy among members [RAP2.2. RAP2.3, RAP2.12, hypoxia responsive ERF1 (HRE1), and HRE2] has been described (Marín-de la Rosa et al., 2014). Therefore, a quintuple mutant defective in all five members, *erfVII*, was additionally included in the analysis (Marín-de la Rosa et al., 2014; Supplementary Figure S3B). To further examine the functional interaction of ORA59 with RAP2.3 and group VII ERFs, we crossed *ora59-1* and *ORA59OE*(#5) with *rap2.3-2* and *erfVII* lines (Supplementary Figures S3B,C). Noticeably, *RAP2.3* expression was much higher in *ORA59OE* plants than in wild type plants (Supplementary Figures S3C), suggesting that *RAP2.3* expression is positively affected by ORA59. However, *ORA59* expression was similar among *ORA59OE* and *ORA59OE rap2.3* lines.

**FIGURE 4:**
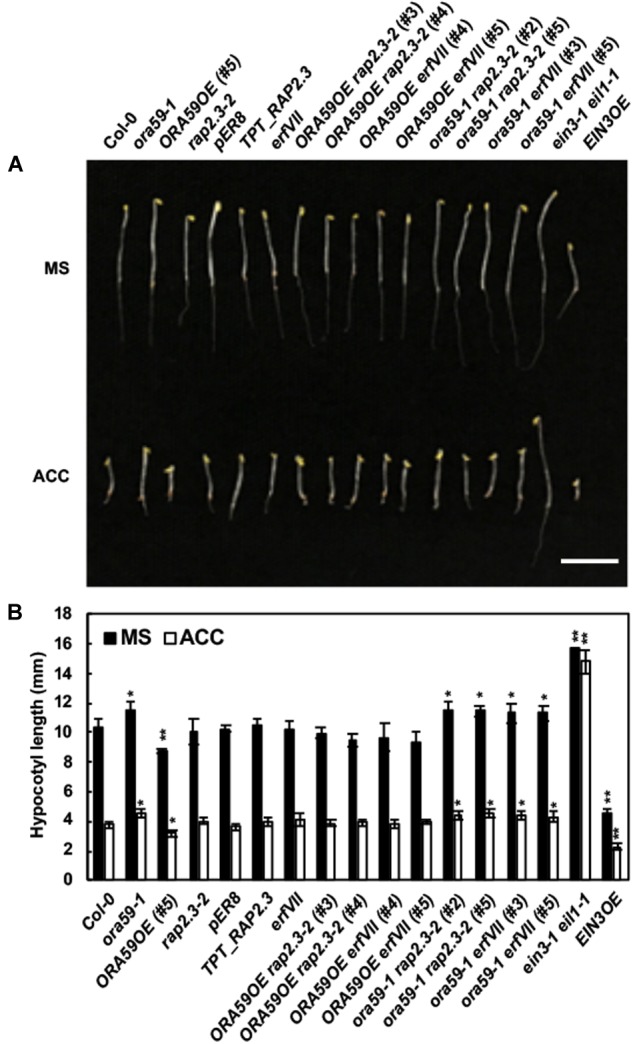
Functions of ORA59 and RAP2.3 in the ethylene response. **(A)** Triple response phenotypes of 4-day-old etiolated seedlings of Col-0, *ORA59* and *RAP2.3* mutants and transgenics, *erfVII*, and their crossed lines in the presence or absence of 10 μM ACC. Bar, 1 cm. **(B)** Hypocotyl lengths of seedlings in **(A)**. The values are means ± SD (*n* = 15). Asterisks indicate significant differences from Col-0 (*t-*test; ^∗^*P* < 0.05; ^∗∗^*P* < 0.01).

Ethylene induces the triple response in seedlings grown in the dark, featured by short, thick hypocotyl, and root, and exaggerated apical hook (Ecker, 1995). We examined whether the triple response is affected by the increased or decreased expression of *ORA59* and *RAP2.3* and determined the hypocotyl length of dark-grown seedlings as a measure of the triple response. In the presence of the ethylene precursor 1-aminocyclopropane-carboxylic acid (ACC), *ORA59OE* seedlings showed the slightly enhanced triple response with a reduction in hypocotyl elongation, and *ora59* mutant showed a weaker triple response (Figure 4). Previously, apical hook formation was impaired in *rap2.3* and *erfVII* mutants (Marín-de la Rosa et al., 2014; Abbas et al., 2015). However, hypocotyl length was not altered in *TPT_RAP2.3*, *rap2.3*, and *erfVII* mutant seedlings. Whereas the enhanced triple response in *ORA59OE* seedlings was abolished in *ORA59OE rap2.3* and *ORA59 erfVII* crossed lines, *ora59 rap2.3* and *ora59 erfVII* mutants displayed hypocotyl elongation similar to *ora59* seedlings. These results suggest that ORA59 at least partly regulates the triple response and that this ORA59 activity depends on RAP2.3.

### ORA59 and RAP2.3 Control Disease Resistance

**FIGURE 5:**
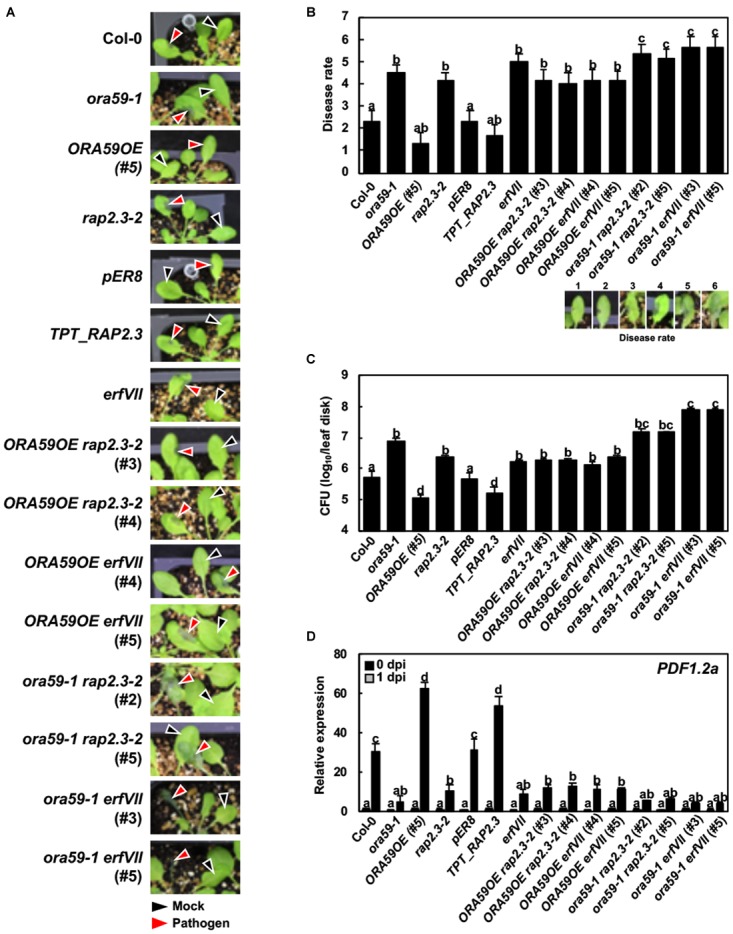
Functions of ORA59 and RAP2.3 in resistance against *P. carotovorum*. **(A)** Disease symptoms in leaves inoculated with *P. carotovorum*. Inoculation sites are indicated by black arrowheads for 0.9% NaCl (mock) and red arrowheads for *P. carotovorum*. **(B)** Disease severity estimations (1–6) in leaves of **(A)** were made. The values are means ± SD (*n* = 6). **(C)** Bacterial growth in leaves inoculated with *P. carotovorum*. The values are means ± SD (*n* = 8). The experiment was repeated three times with similar results. **(D)** Expression of *PDF1.2a* in leaves inoculated with *P. carotovorum*. The values represent means ± SD from three independent experiments. Five-week-old plants were treated with 10 μL of *P. carotovorum* at 10^5^ cfu/mL **(A,B)** and 10^4^ cfu/mL **(C,D)** for 1 day. Different letters indicate statistically significant difference (Tukey’s HSD test; *P* < 0.05).

The ethylene signaling pathway regulates resistance against necrotrophic pathogens (Kim et al., 2013). In a previous report, *ORA59* was involved in resistance to the necrotrophic fungal pathogen *B. cinerea* but not to the other fungus *A. brassicicocla* in Arabidopsis (Pré et al., 2008). To further assess the roles of ORA59 and RAP2.3 in disease resistance, the prepared *ORA59*, *RAP2.3*, and *ERFVII* lines were challenged with the necrotrophic bacterial pathogen *P. carotovorum*. *P. carotovorum* causes soft rot disease in plants through secretion of cell wall-degrading enzymes such as pectinases and cellulases (Norman-Setterblad et al., 2000). The bacteria replicate feeding on materials released from injured cells. The severity of necrotic lesions and bacterial growth were scored and dead cells in lesions were stained with trypan blue. Necrotic disease symptoms developed in Col-0 plants, which were decreased in *ORA59OE* and *TPT_RAP2.3* plants but increased in *ora59* and *rap2.3* mutants (Figures 5A–C and Supplementary Figures S4, S5). This demonstrates that ORA59 and RAP2.3 positively regulate resistance to *P. carotovorum*. Disease severity was similar in *rap2.3* and *erfVII* plants, suggesting that RAP2.3 plays a major role in disease resistance among the group VII ERF members. When disease symptoms were compared between *ORA59OE* and *ORA59OE rap2.3*, and *rap2.3* lines, the increased resistance in *ORA59OE* plants was compromised in *ORA59OE rap2.*3 plants, which showed susceptibility similar to *rap2.3* mutant. The susceptibility to *P. carotovorum* was further increased in *ora59 rap2.3* and *ora59 erfVII* plants. *PDF1.2a* expression was highly induced in wild type and *ORA59*- and *RAP2.3*-overexpressing plants by *P. carotovorum* infection, but largely decreased in mutants and crossed lines with susceptible phenotypes (Figure 5D). There was no difference in *PDF1.2a* expression among untreated plant lines. These results suggest that ORA59 interacts with RAP2.3 in both dependent and additive manners to induce resistance to *P. carotovorum*.

## Discussion

Ethylene, as one of stress hormones, regulates defense responses in plants (van Loon et al., 2006; Kwon et al., 2009; Broekgaarden et al., 2015). Ethylene signaling ultimately leads to modulation of defense gene expression, which includes the action of ERFs (Müller and Munné-Bosch, 2015). ORA59 is a group IX ERF transcription factor that regulates the expression of JA- and ethylene-dependent genes including *PDF1.2* by binding to the GCC-box, a *cis*-acting ethylene response element (Pré et al., 2008; Zarei et al., 2011). In this study, we investigated the role of ORA59 in plant defense. Y2H screening led to the identification of 12 putative ORA59 interactors, none of which have been characterized in association with ethylene and/or pathogenic responses except for RAP2.3. RAP2.3 belongs to the group VII ERF family composed of RAP2.2, RAP2.3, RAP2.12, HRE1, and HRE2 (Nakano et al., 2006). Previous studies demonstrate that the common roles of group VII ERF transcription factors are associated with plant survival under hypoxia/anoxia and other stress conditions (Giuntoli and Perata, 2018). HRE1 and HRE2 positively affected anaerobic responses by increasing anaerobic gene expression and ethanol fermentation (Licausi et al., 2010). Overexpression of *RAP2.2*, *RAP2.3*, and *RAP2.12* conferred tolerance to multiple abiotic stresses including hypoxia, whereas each single and *erfVII* quintuple mutants showed hypersensitive stress responses (Hinz et al., 2010; Bui et al., 2015; Papdi et al., 2015; Vicente et al., 2017). Furthermore, *erfVII* mutant plants had defects in the response to the gall-forming pathogen *Plasmodiophora brassicae* and in the stomatal immune response to the hemobiotrophic pathogen *P. syringae* pv *tomato*, suggesting their roles in biotic stress responses (Gravot et al., 2016; Vicente et al., 2018). According to recent findings, an increased oxygen level induces Cys oxidation and subsequent arginylation at the N-terminus of group VII ERFs, leading to proteolysis via the N-end rule pathway (Gibbs et al., 2011; Licausi et al., 2011). These proteins accumulate under low oxygen conditions, and in this manner, act as oxygen sensors, regulating hypoxic responses. It was previously shown that RAP2.12 binds to acyl-CoA-binding proteins ACBP1/ACBP2 to protect it from the N-end rule pathway in air (Licausi et al., 2011). This may explain our observation of RAP2.3-GFP/RFP proteins in protoplasts, as had been also demonstrated for the interaction of RAP2.3 and ACBP2/ACBP4 (Li and Chye, 2004; Li et al., 2008). CaMV 35S-driven constitutive expression of *RAP2.3* may additionally contribute to RAP2.3 accumulation in protoplasts.

In this study, *TPT_RAP2.3*, *rap2.3*, and *erfVII* mutant seedlings showed the hypocotyl response to ethylene similar to wild type, although ORA59 required RAP2.3 for ethylene responses. On the other hand, previous studies have addressed the involvement of group VII ERFs in ethylene responses (Zhao et al., 2012; Marín-de la Rosa et al., 2014). When treated with ACC, *rap2.2 rap2.12* double mutants exhibited a decrease in hypocotyl length, whereas their single mutants showed no differences in the triple response from wild type seedlings (Zhao et al., 2012). In addition, partial defects in apical hook formation were observed in *rap2.3*, *rap2.12*, and *erfVII* mutants in response to combined treatments of ACC and GA, but not to ACC alone (Marín-de la Rosa et al., 2014). Additional functional analyses of group VII ERF members are needed to clarify their specific and overlapping roles in ethylene responses.

A previous study demonstrates that ORA59 plays a role in disease resistance (Pré et al., 2008). ORA59-overexpressing and silenced plants were tested for responses to the necrotrophic fungal pathogens *B. cinerea* and *A. brassicicola*. Here we additionally assessed the role of ORA59 in the response to *P. carotovorum*. Previous and our data suggest that ORA59 positively regulates resistance to *B. cinerea* and *P. carotovorum*. In contrast, resistance to *A. brassicicola* was not affected in *ORA59*-silenced plants compared to wild type plants. The Columbia ecotype Col-0 plants are susceptible to *B. cinerea* and *P. carotovorum*, but resistant to *A. brassicicola* (Thomma et al., 1998, 1999; Norman-Setterblad et al., 2000; Pré et al., 2008), suggesting that ORA59 may be a component of basal immunity.

**FIGURE 6:**
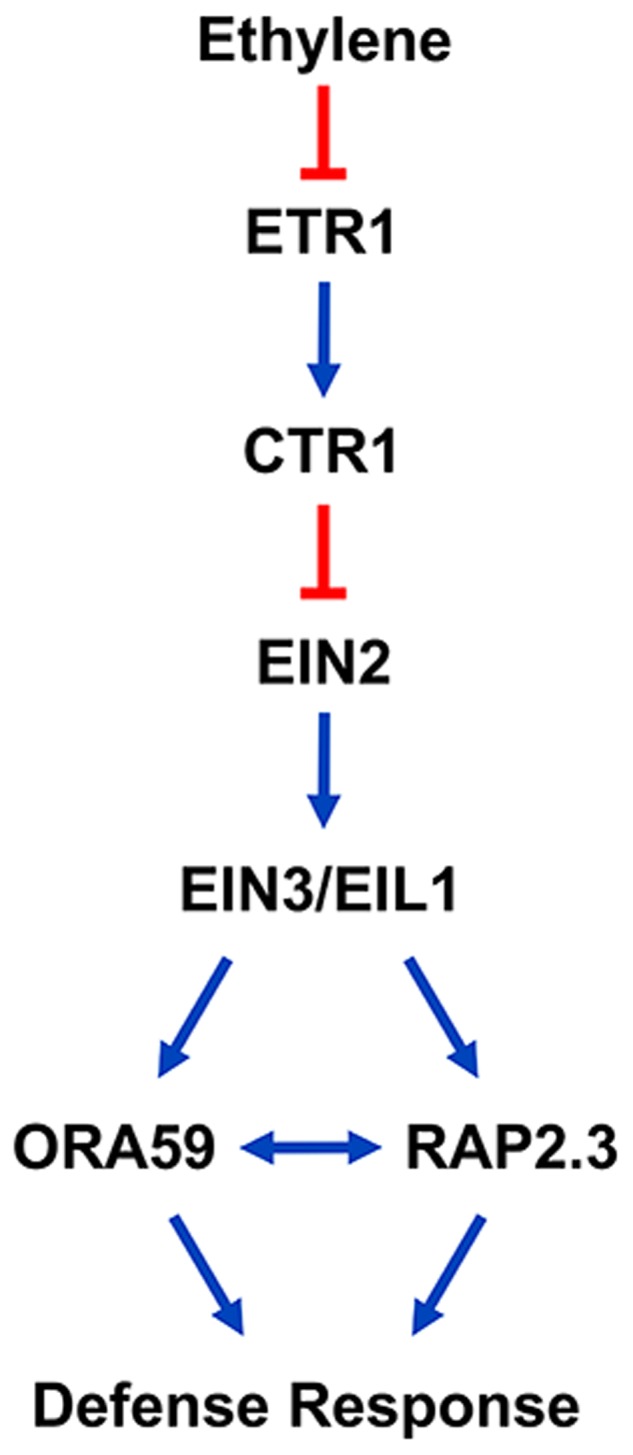
A model proposing that ORA59 and RAP2.3 interact downstream of EIN2 and EIN3/EIL1 in the ethylene signaling pathway to regulate defense responses. Arrows and bars indicate positive and negative regulation, respectively, and double arrow indicates the interaction between ORA59 and RAP2.3.

ORA59 and RAP2.3 share common features associated with ethylene responses, as demonstrated in previous reports. The expression of *ORA59* and *RAP2.3* was induced by ACC and ET, but greatly decreased in the *ein2-1* mutant background (Büttner and Singh, 1997; Ogawa et al., 2005; Li et al., 2008; Pré et al., 2008). Here we also showed that ET-induced *ORA59* and *RAP2.3* expression is abolished in *ein2* and *ein3 eil1* mutants. These gene expression patterns indicate that ORA59 and RAP2.3 are located downstream of EIN2 and EIN3/EIL1 in ethylene signaling. In fact, *RAP2.3* was identified as an EIN3 target in a genome-wide study of EIN3-regulated genes using chromatin immunoprecipitation followed by sequencing (Chang et al., 2013). In this study, subcellular localization of ORA59 and RAP2.3 was additionally regulated by ET. They both translocated to the nucleus in response to ET treatment. These results suggest that the transcriptional activities of ORA59 and RAP2.3 are regulated by ethylene signaling through several mechanisms, including gene expression, protein stability, and nuclear localization. On the other hands, hormone responses of these two genes were somewhat different. Whereas *ORA59* expression was mostly specific to ET, JA, and SA, *RAP2.3* responded to all the tested hormones, ET, JA, SA, ABA, and GA. This may reflect that RAP2.3 is multifunctional in regulating development and various stress responses, as previously reported. *RAP2.3* overexpression conferred tolerance to hypoxia, heat, and oxidative and osmotic stresses (Ogawa et al., 2005; Papdi et al., 2015). Regarding its role in development, RAP2.3 positively regulated apical hook development, and this activity was suppressed through the interaction with the DELLA protein GIBBERELLIN INSENSITIVE, a master negative regulator in GA signaling (Marín-de la Rosa et al., 2014). ORA59 and RAP2.3 directly bound to the GCC box and their overexpression increased *PDF1.2* expression (Ogawa et al., 2005; Pré et al., 2008; Zarei et al., 2011). *ORA59* overexpression enhanced resistance to *B. cinerea*, and *RAP2.3* expression was induced by *B. cinerea* infection (Li et al., 2008; Pré et al., 2008). Previous studies and our data together suggest that ORA59 and RAP2.3 play common roles in ethylene-mediated defense responses (Figure 6).

In general, transcription factors form dimers with identical proteins, other members in the same family or proteins in different gene families to bind DNA and regulate gene expression. Interacting partners are selected in the context of developmental stages and stress responses, determining the specificity for target genes and leading to a specific cellular event. To date, several transcription factors that interact with ORA59 and RAP2.3 have been reported. He et al. (2017) demonstrated that ORA59 interacts with EIN3, and this interaction is required for SA-mediated reduction in ORA59 protein levels. RAP2.3 was associated with DELLA proteins (Marín-de la Rosa et al., 2014). In this case, DELLAs prevented RAP2.3 binding to the promoter of its target genes and counteracted the activity of RAP2.3 promoting apical hook closing. There was also a report about the interaction between RAP2.3 and the bZIP transcription factor TGA4, suggesting their synergistic functions to regulate gene expression during the defense response (Büttner and Singh, 1997). Our findings reveal the case of binding between members belonging to the same ERF family and imply the possibility of combinatorial interactions of ERFs for diverse gene expression regulation. Further studies of the interactions among ERFs and other types of transcription factors will enhance our understanding of the complexity of gene expression and defense responses.

## Author Contributions

NK and OP designed the research, analyzed the data, and wrote the article. NK and YJ performed the research.

## Conflict of Interest Statement

The authors declare that the research was conducted in the absence of any commercial or financial relationships that could be construed as a potential conflict of interest.
