# Corrigendum: AP2/ERF Family Transcription Factors ORA59 and RAP2.3 Interact in the Nucleus and Function Together in Ethylene Response

**DOI:** 10.3389/fpls.2019.00042

**Published:** 2019-01-30

**Authors:** Na Young Kim, Young Jin Jang, Ohkmae K. Park

**Affiliations:** Department of Life Sciences, Korea University, Seoul, South Korea

**Keywords:** *Arabidopsis thaliana*, ORA59, RAP2.3, ethylene response factor, ethylene, *Pectobacterium carotovorum*, disease resistance, plant immunity

In the original article, there was a mistake in Figure 1B as published. In the input (α-His) panel, lanes 3 and 4 have been changed. The corrected Figure 1B appears below.

**Figure 1:**
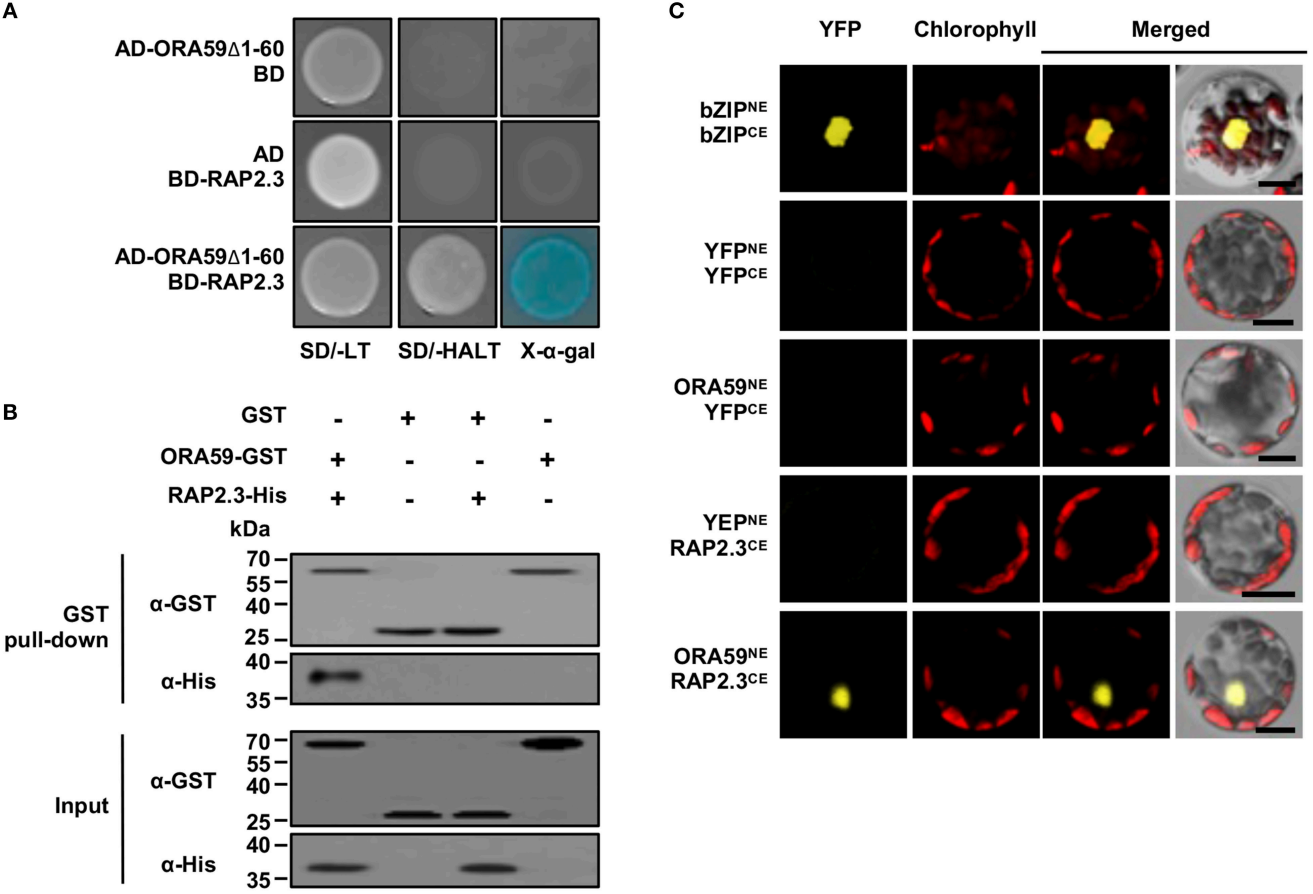
Physical interaction of ORA59 with RAP2.3. **(A)** Yeast two-hybrid assay. ORA59 with N-terminal 60 amino acids deleted (ORA59Δ1-60) and full-length RAP2.3 were fused with GAL4 AD and BD, respectively. Their interactions were tested on selective media SD/-AHLT and in the presence of X-α-Gal. **(B)** *in vitro* GST pull-down assay. GST or ORA59-GST was incubated with RAP2.3-His and precipitated with glutathione sepharose 4B beads. Proteins were detected by immunoblotting with anti-GST and anti-His antibodies. Input shows 1% of the amount used in binding reactions. WB, western blotting. **(C)** BiFC assay. YFP^NE^, YFP^CE^, and their fusion proteins bZIP63^NE^, bZIP63^CE^, ORA59^NE^, and RAP2.3^CE^ were expressed in Arabidopsis protoplasts as indicated. YFP fluorescence signals were visualized under a confocal microscope. Bars, 10 μm. Experiments were repeated three times with similar results.

The authors apologize for this error and state that this does not change the scientific conclusions of the article in any way. The original article has been updated.

## Conflict of Interest Statement

The authors declare that the research was conducted in the absence of any commercial or financial relationships that could be construed as a potential conflict of interest.

