# Supplementary material for: AP2/ERF Family Transcription Factors ORA59 and RAP2.3 Interact in the Nucleus and Function Together in Ethylene Responses
Source: Front Plant Sci. 2018 Nov 19;9:1675. doi: 10.3389/fpls.2018.01675 (PMC6254012; doi:10.3389/fpls.2018.01675)
Supplement: Supplementary file 1 [file Table_1.pdf]

Table S1. Primers used in this study.

AGI code	Gene name	Forward primer	Reverse primer
qRT-PCR			
AT1G06160	*ORA59*	GATCAGGCGGCTTTCGCTT	CAGCACCTAAATCCTCAAGAACC
AT3G14230	*RAP2.2*	GCGTCGTATCCCAGAAACG	CAAGGCGTTGTCAAGGTATGC
AT3G16770	*RAP2.3*	CCATCCCACCAACCAAGTTAACG	CATCAGCAGTCCAATGCGA
AT1G53910	*RAP2.12*	TGCTAGAGCTTACGATGCTG	GAAGACTCCTCCAATCATGG
AT1G72360	*HRE1*	CTCAATTTCCCTAACGAATCCTCT	CCGTAAAAGGCATCTGCGAGAA
AT2G47520	*HRE2*	ATGTGTGGGGGAGCTATCATTT	ATTGGAGTCTTGATAGCTCCAT
AT3G23240	*ERF1*	CGGCTTTAGCCTACGATC	CTCCTCAAGGTACTGTTCTC
AT5G44420	*PDF1.2a*	GCTAAGTTTGCTTCCATCATCACCC	CATGGGACGTAACAGATACACTTGTGT
AT2G37620	*ACTIN1*	GGCGATGAAGCTCAATCCAAACG	GGTCACGACCAGCAAGATCAAGACG
Genotyping			
AT1G06160	*ORA59*	AAGGGATAAGAGTGTGGCTTGGGA<br>ATGCTGCAGACGGTAACAAAC	GTAGAAGCAGAGATTATCAAGAACA
