# Supplementary material for: AP2/ERF Family Transcription Factors ORA59 and RAP2.3 Interact in the Nucleus and Function Together in Ethylene Responses
Source: Front Plant Sci. 2018 Nov 19;9:1675. doi: 10.3389/fpls.2018.01675 (PMC6254012; doi:10.3389/fpls.2018.01675)
Supplement: Supplementary file 2 [file Image_1.pdf]

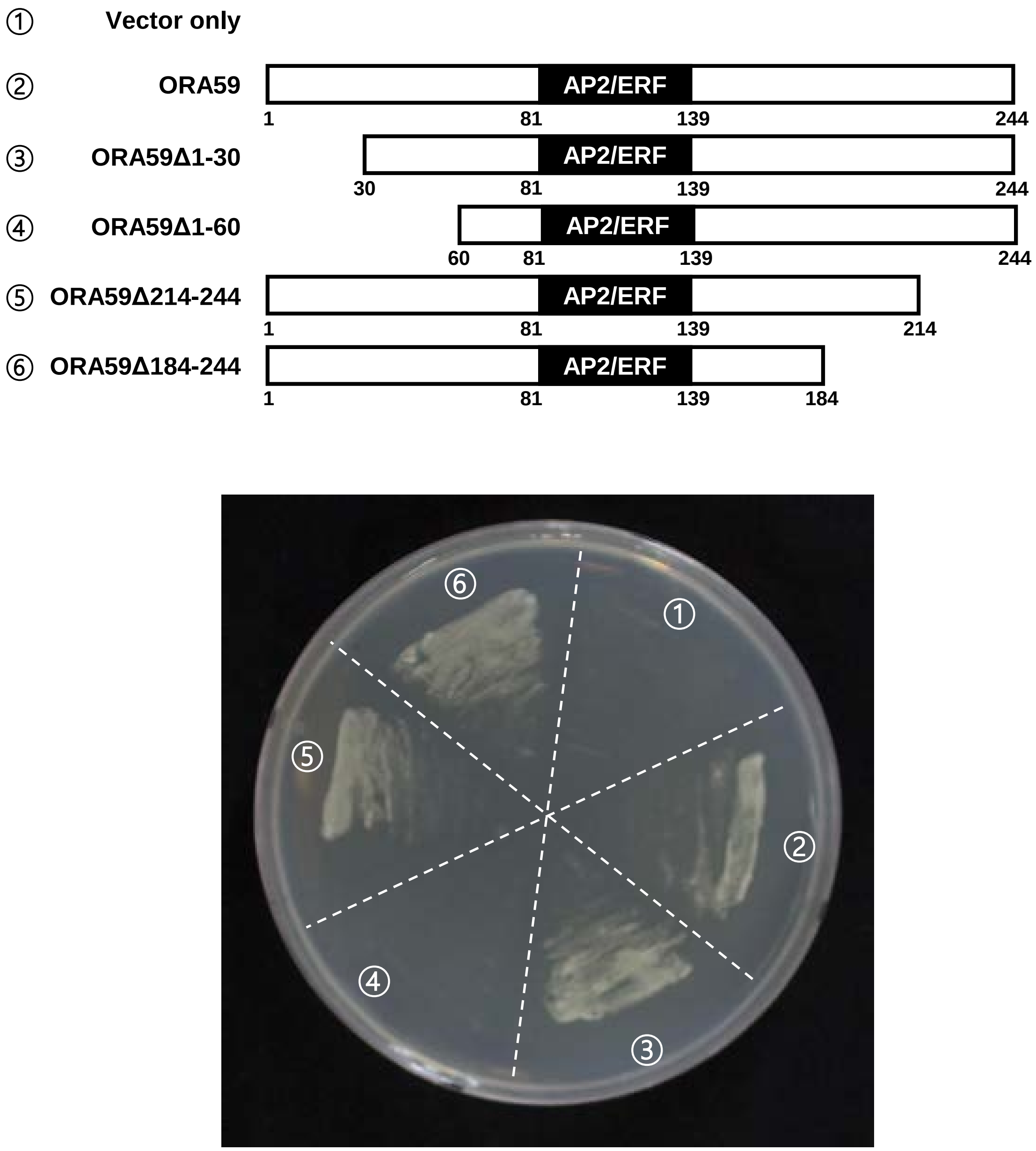

Figure S1. Yeast two-hybrid assay for autoactivation of truncated forms of ORA59. The full-length ORA59 (2) and truncated forms with N-terminal 30 (3) and 60 (4) amino acids deleted and with C-terminal 30 (5) and 60 (6) amino acids deleted were tested for autoactivation on SD/-AHLT medium.
