# Supplementary material for: AP2/ERF Family Transcription Factors ORA59 and RAP2.3 Interact in the Nucleus and Function Together in Ethylene Responses
Source: Front Plant Sci. 2018 Nov 19;9:1675. doi: 10.3389/fpls.2018.01675 (PMC6254012; doi:10.3389/fpls.2018.01675)
Supplement: Supplementary file 3 [file Image_2.pdf]

Kim et al., Figure S2

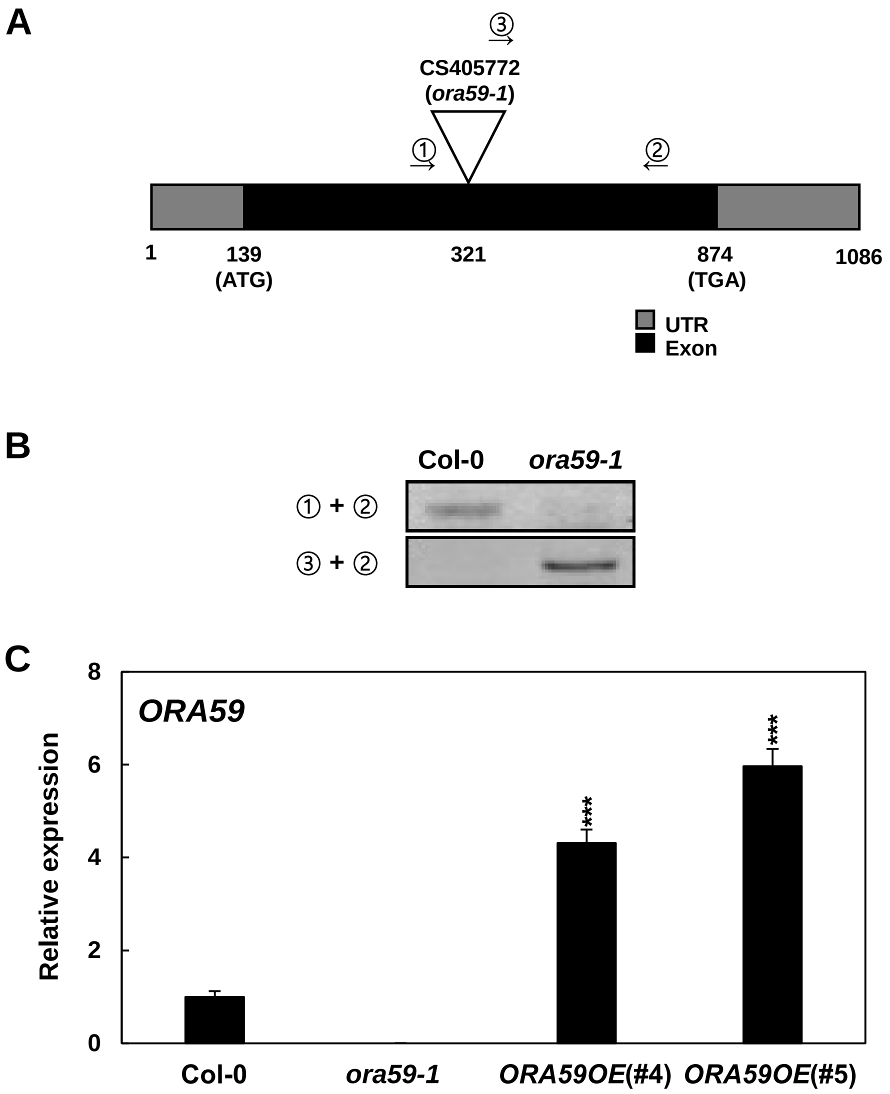

Figure S2. Preparation of *ora59* and *ORA59OE* lines. **(A)** Genomic structure of *ORA59* showing the positions of T-DNA insertion. gDNA sequences are represented by a single exon (black box) and untranslated regions (UTRs) (gray boxes). Numbers refer to nucleotides of *ORA59* gene. Arrows indicate primers used for PCR. **(B)** Genotyping PCR using primers indicated in **(A)** verified homozygous T-DNA insertions. **(C)** *ORA59* expression in Col-0, *ora59-1*, and *ORA59OE* lines. Results represent means (±SD) of 3 biological replicates. Asterisks indicate significant differences from Col-0 (*t* test; ***P < 0.001).
