# Supplementary material for: AP2/ERF Family Transcription Factors ORA59 and RAP2.3 Interact in the Nucleus and Function Together in Ethylene Responses
Source: Front Plant Sci. 2018 Nov 19;9:1675. doi: 10.3389/fpls.2018.01675 (PMC6254012; doi:10.3389/fpls.2018.01675)
Supplement: Supplementary file 4 [file Image_3.pdf]

Kim et al., Figure S3

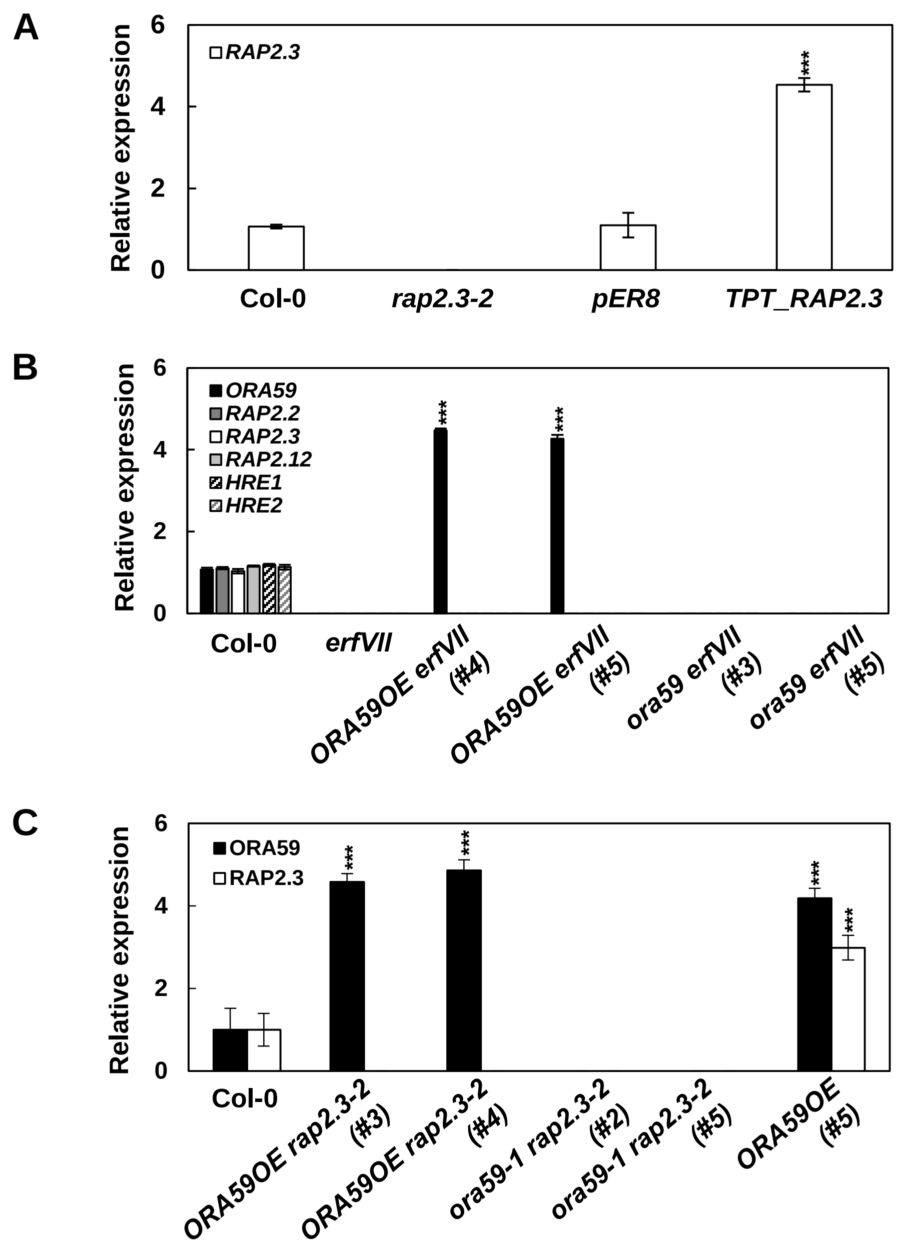

Figure S3. Preparation of *rap2.3*, *TPT_RAP2.3*, *erfVII*, and crossed lines. **(A)** *RAP2.3* expression in *rap2.3* and *TPT_RAP2.3* plants. *pER8* and *TPT_RAP2.3* plants were treated with 10 μM β-estradiol for 6 h before harvest. **(B)** Expression of *ORA59* and group VII ERF genes in *erfVII*, *ORA59OE erfVII*, and *ora59 erfVII* lines. **(C)** Expression of *ORA59* and *RAP2.3* in *ORA59OE rap2.3, ora59 rap2.3, and ORA59OE* lines. Results represent means (±SD) of 3 biological replicates. Asterisks indicate significant differences from Col-0 (*t* test; ****P* < 0.001).
