# Supplementary material for: AP2/ERF Family Transcription Factors ORA59 and RAP2.3 Interact in the Nucleus and Function Together in Ethylene Responses
Source: Front Plant Sci. 2018 Nov 19;9:1675. doi: 10.3389/fpls.2018.01675 (PMC6254012; doi:10.3389/fpls.2018.01675)
Supplement: Supplementary file 5 [file Image_4.pdf]

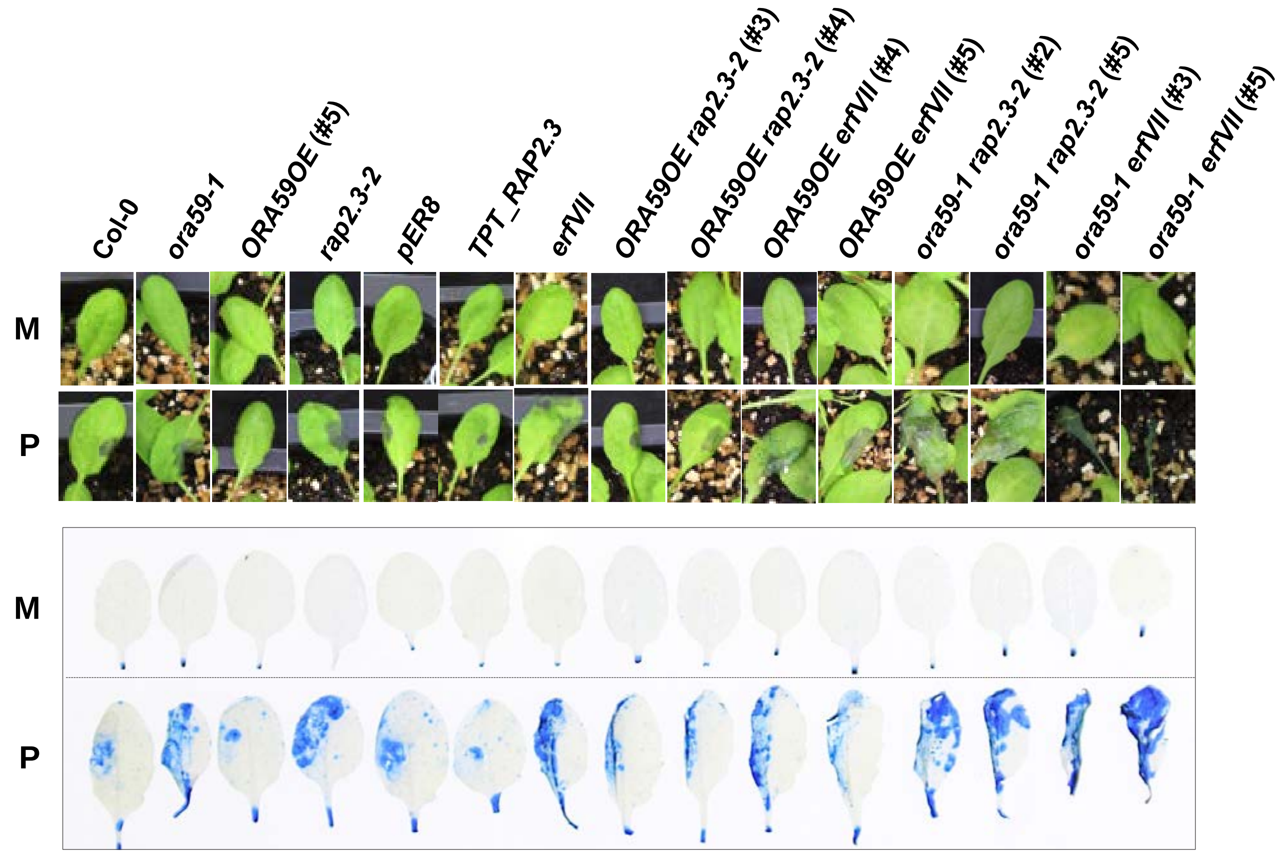

Figure S4. Disease Development in leaves inoculated with *P. carotovorum*. Enlarged views of disease symptoms shown in **Figure 5A** (upper) and necrotic lesions in leaves stained with trypan blue (lower). Three-week-old plants were treated with 10 µL of *P. carotovorum* at 10^6 cfu/mL for 1 day. M, mock treatment; P, pathogen treatment.
