# Supplementary material for: AP2/ERF Family Transcription Factors ORA59 and RAP2.3 Interact in the Nucleus and Function Together in Ethylene Responses
Source: Front Plant Sci. 2018 Nov 19;9:1675. doi: 10.3389/fpls.2018.01675 (PMC6254012; doi:10.3389/fpls.2018.01675)
Supplement: Supplementary file 6 [file Image_5.pdf]

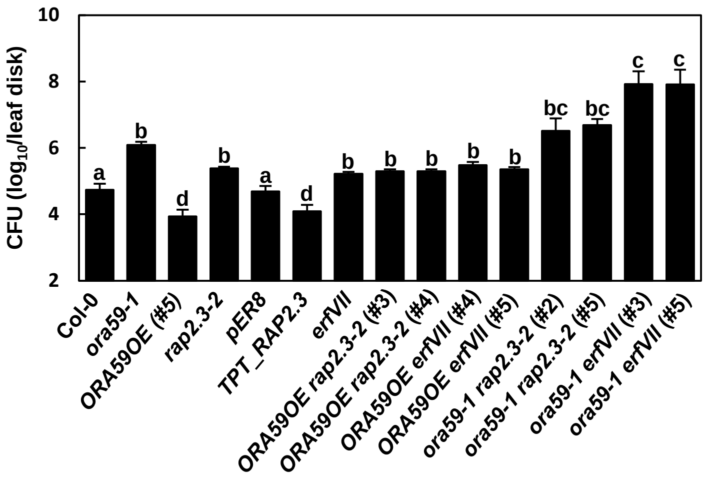

Figure S5. Bacterial growth in leaves inoculated with *P. carotovorum*. The values are means ± SD (n = 8). This is a biological repeat of bacterial growth analysis in **Figure 5C**. Different letters indicate statistically significant difference (Tukey's HSD test; $P < 0.05$).
